# Representation in Health Professions Education: Striving for an Inclusive Health Professions Education Community

**DOI:** 10.5334/pme.883

**Published:** 2023-10-26

**Authors:** Zakia Dimassi, Halah Ibrahim

**Affiliations:** 1 Practice of Medicine, United Arab Emirates; 2 Physicianship, Khalifa University College of Medicine and Health Sciences, Abu Dhabi, United Arab Emirates; 3 Khalifa University College of Medicine and Health Sciences, Abu Dhabi, United Arab Emirates

## Abstract

Author representation and inclusivity in health professions education (HPE) scholarship is receiving increasing attention in academic medicine, with multiple articles calling for greater equity related to gender, geographic, and institutional affiliations. Despite journal efforts to seek diversity, authors from high-income English-speaking countries are the most highly represented in HPE scholarship. Less attention, however, has been focused on the openness of medical education scholars, themselves, to engaging in international collaborations with authors and institutions from low-and-middle income countries. This eye-opener is inspired by the authors’ personal experiences in HPE scholarship from an international medical educator perspective and advocates for the creation of an open and inclusive multinational medical education community. We offer suggestions that can help create opportunities for networking, collaboration, and promoting a sense of belonging among HPE scholars worldwide. As researchers, journal editors and associate editors, and faculty in HPE programs, we can work together to create a welcoming and accommodating environment that embraces non-dominant voices and perspectives, with the ultimate goal of achieving diversity and equity in HPE scholarship.

Health professions education (HPE) is a fast-growing discipline that has recently undergone major paradigm shifts in curricular design, assessment models, and regulatory requirements, with a rapid increase in the volume of research generated [1,2]. A globalization movement has also ensued. Spurred by the migration of patients, healthcare professionals and medical trainees across international borders, there is a growing worldwide interconnection of medical schools and academic medical centers, with their curricula, practices and values [3]. Concomitantly, outcries against inequity and exclusion in healthcare and HPE are on the rise, heightened by events that marked the COVID-19 pandemic [4,5,6]. These developments should have stipulated efforts to promote international collaborations that involve a more diverse pool of HPE scholars. Yet, the HPE publication landscape remains gravely skewed toward high-income countries in North America and Europe [7]. such that the quality and relevance of submitted articles from other regions of the world may not be determining factors for publication [8,9]. While top medical education journals claim to reflect global perspectives, to date, authorship in medical education scholarship is substantially less diverse than in many other fields, including education, medicine and biologic sciences [7,9], with relatively little change over the past decade [7,8]. In this manuscript, we describe barriers faced by international researchers from the perspectives of Global South authors. We also share our personal experiences and offer recommendations for creating a more inclusive HPE community.

In the backdrop of the current reality in medical education and HPE publication are two phenomena that pervade many aspects of healthcare globally: colonialism and Northern elitism. Western medicine was a key component of the colonial endeavor that positioned itself as the sole source of scientific truths and enlightenment [10], and medical education has followed suit [11]. For example, Western-based medical education accreditation standards and best practices are commercially exported to the rest of the world [12,13], prompting a vocal warning from international educators, who emphasize the importance of addressing context and diverse local needs in medical education [3,14]. Similarly, recent attention has focused on the recognition and dismantling of the Western predominance in HPE scholarship. Naidu argues for a “decolonial praxis,” involving awareness, deliberation and action, to ultimately shift the power structure in health professions research [4]. Kusurkar describes the phenomenon as a “leaky pipeline” of medical education publication practices, whereby multiple systemic factors, including the lack of reviewer and editorial board diversity, prevent knowledge generated in the Global South from successful publication in major medical education journals [5]. She describes her personal experience of overcoming a hierarchal system dominated by Western authors and ideologies. Mokhachane, Green-Thompson, and Wyatt use personal narratives to describe how researchers from the Global South are systematically excluded from publication in HPE journals [6]. Through the metaphor of a slave plantation in the American South in the 1800s, they portray the power imbalances that Global South authors must overcome in order to be invited into the HPE research community [6].

It is evident that medical education scholarship has fallen far short of fulfilling its social and ethical obligations of ensuring equity, human development, capacity building, and diversity [15]. Multi-institution collaborations are generally formed within the same geographic region, and research networks are dominated by authors from elite institutions across North America and Europe [8]. Moreover, partnerships and international collaborations with underrepresented regions have been plagued by power imbalances [15]. So, critical questions remain unanswered. Why do the HPE scholarship and literature remain so exclusive? Why are opportunities not created to engage educators from less represented regions of the world? Although historical inequities and systems of oppression may partially address these questions, other dynamics are involved. Non-Western scholars confront personal barriers. Specifically, informal networks of HPE researchers have created exclusive communities that require particular rites of passage. Indeed, the increasing professionalization of medical education over the past several decades requires an advanced degree in HPE and published evidence of scholarship to demonstrate accomplishment in the field [1,2]. Professionals who succeed in meeting these criteria are invited to present at national conferences and earn prestigious seats on journal editorial boards. Not only are their voices amplified, they also assume the privileged roles of selecting issues of relevance and determining whose voices are also heard. Examining the medical education publication landscape through a social identity theory lens, these well-established researchers are akin to the “in-group,” leaving outsiders marginalized, with little accessibility to the privileges and perks of academic publishing [16]. The social capital gained through this networking facilitates access to information that is not readily available to the outsider, including stylistic preferences of journal editors and the “hot topics” that are more likely to get published.

Our personal experiences have highlighted the struggles of the HPE outsiders. We both work in international medical education. To boost our careers and research output, we completed master’s degrees in HPE from renowned institutions that boast international student cohorts and collaborations. Upon graduation, however, we had different experiences with the HPE community. One sought networking with HPE gurus through social media platforms (Twitter, LinkedIn), but the minimal responsiveness felt dismissive and imparted a sense of disillusionment. The other author was fortunate to join multinational research collaborations, gain positions on journal editorial boards, and now advocates for greater geographic diversity in HPE scholarship. Despite these different experiences and outcomes, there is a uniform message: the medical education scholarship community can and should make a deliberate choice to be open and inclusive. As researchers, journal editors and associate editors, and faculty in HPE programs, it is the responsibility of every member of the medical education community to actively engage colleagues from diverse backgrounds. Just as we mentor trainees and junior faculty and explicitly support inclusivity and diversity, it is incumbent on us to support authors from countries underrepresented in the medical education landscape, particularly through scholarship collaborations. Providing international educators with opportunities to build professional networks supports personal and professional growth and can create a sense of competence and belonging within the HPE community [16,17]. Moreover, creating a welcoming and accommodating environment that embraces non-dominant voices and perspectives not only helps us in achieving equity and antiracism in HPE scholarship, it also enriches scholarly output by increasing topical diversity in the medical education literature [9,12]. It is admittedly difficult to attain this goal in a fast-paced world that is, paradoxically, both wide open to connection and communication, yet overburdened with hurdles that limit our ability to connect and communicate. Cultural humility is paramount in these collaborations. It is important to focus on collaborative and capacity-building research agendas that address local priorities, while availing local researchers the opportunity for prominent authorship positions. Dismantling deeply entrenched power hierarchies and structural barriers to shift the power in HPE scholarship is a substantial undertaking that will take time and considerable socio-political capital, but right now, the HPE research community can choose to become a more inclusive space.

Avenues for improving the current state of affairs in the HPE community exist (See Figure 1), and we must proceed by harvesting the low-hanging fruit, including improving access and reach to medical education conferences. For international educators and scholars, the large time and financial commitments and visa requirements may prohibit attending meetings that can provide opportunities for networking, scholarly collaboration, and a sense of community. Adopting the available electronic platforms and optimizing them to enhance the virtual attendance experience will help overcome these obstacles. A successful conference experience can enable educators to disseminate their work and can serve as a nucleus to forge long-lasting collaborations that would lead to career advancement. An online platform alone, however, does not ensure that all groups can attend and meaningfully participate. Creating an inclusive and diverse conference must be intentional by design, and starts with a conference organizing committee that represents different genders, ethnicities, geographic regions, career stages, and other aspects of diversity. Additional strategies to engage international educators include building an inclusive communication strategy with the aim of reaching a broader audience, diversifying thematic sessions, and prioritizing individuals from underrepresented groups to chair sessions and give plenary and keynote speeches [18].

**Figure 1:**
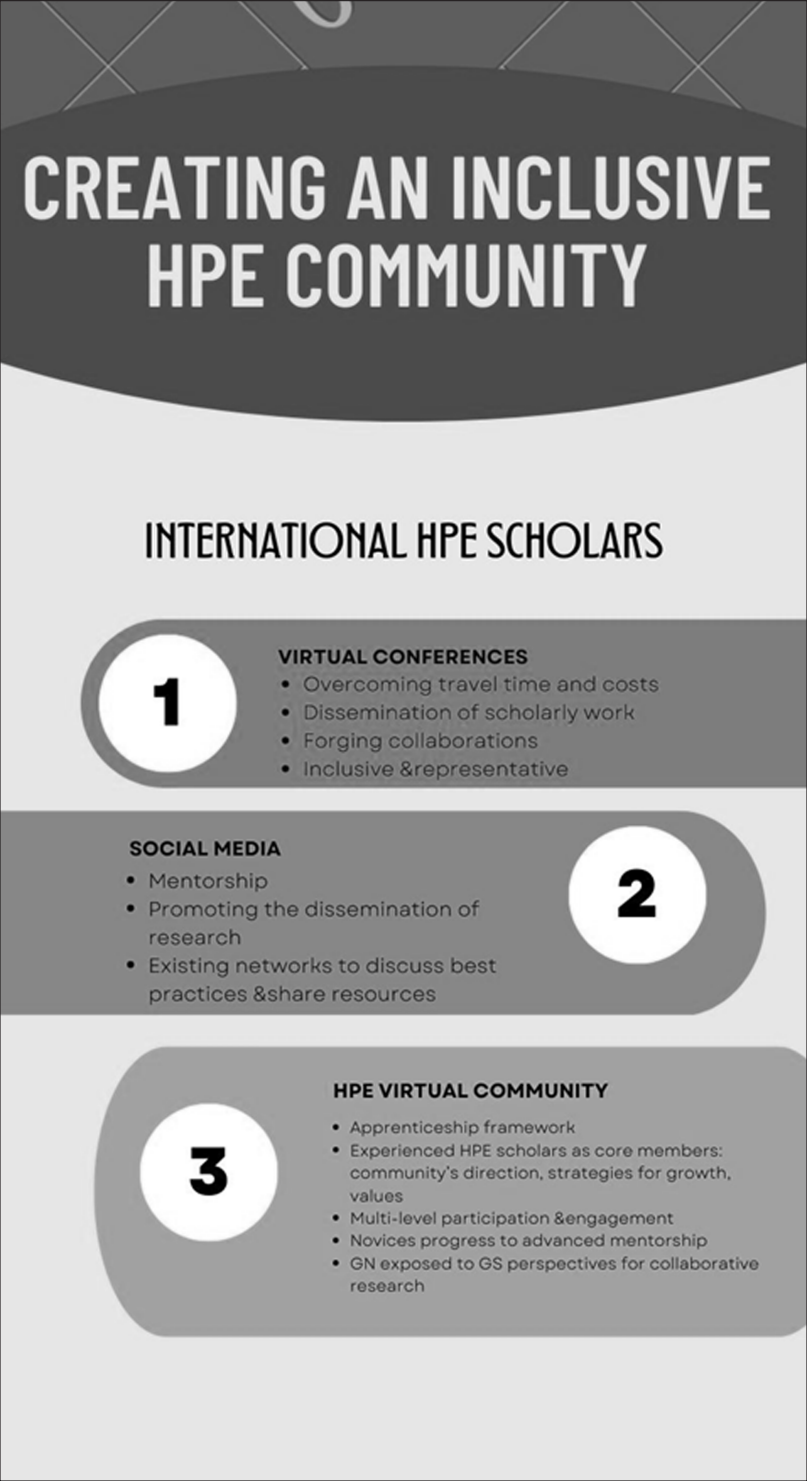
Recommendations for creating a more inclusive health professions education (HPE) community. Abbreviations: GN = global north; GS = global south.

The online realm is also a feasible and accessible tool to forge collaborations, particularly with underrepresented parts of the world, through the development of virtual communities of practice. Virtual communities can provide access to a larger community with shared interests. Unrestrained by geographic distance or time zones, virtual communities can provide opportunities for very much needed mentorship and support, spark innovation, and lead to robust multinational research projects [19]. Directors of HPE programs can take a lead role in fostering international collaborations among students and graduates of their programs by maintaining online directories of graduates and their research interests. Strong collaborative communities can continue even after the degree is awarded. Social media platforms, such as Twitter or Instagram, can also serve as powerful tools to unite and maintain communities. Studies have shown the role of Twitter, for example, in creating mentorship opportunities and promoting the dissemination of research for women physicians [20,21]. Global South scholars can leverage existing networks to connect with individuals and organizations to engage in discussions about best practices in HPE scholarship, share resources, and identify opportunities to collaborate on research projects.

Given the Global North’s extractive history, it may be preferable for international HPE scholars to intentionally create their own diverse and inclusive communities. Organizing and maintaining a virtual community in HPE scholarship can be a daunting task, especially if there is no clear leadership or ownership structure in place. Yarris and colleagues provide an easily adaptable apprenticeship framework for a virtual community aimed to support medical education scholarship [19]. A committee comprised of experienced scholars from diverse backgrounds forms the core members of the community and is responsible for setting the community’s direction, managing the core knowledge, such as organizing the Twitter chats, developing strategies for growth, and ensuring that the community’s values are upheld [19]. This model allows for various levels of participation and engagement of other members, depending on individual interest and experience. It also allows for novice researchers to gain valuable experience and the opportunity to progress to core membership [22]. An additional benefit of such a community is that it will enable interested Global North members to be exposed to new perspectives and to find Global South partners for collaborative research projects.

The ultimate goal of these strategies is the creation of accommodating, open, and inclusive spaces where all educators can actively participate in setting the research agenda [23]. ZD and HI were fortunate to meet and work together, but what about other educators who feel isolated and not heard? It is our collective responsibility to help them to find their voices.
